# Changes in the distribution of occlusal forces in the course of the orthodontic retention phase: A prospective cohort study

**DOI:** 10.1007/s00056-023-00480-4

**Published:** 2023-06-29

**Authors:** F. Fritz, N. Daratsianos, C. Bourauel, Spyridon N. Papageorgiou, A. Jäger

**Affiliations:** 1 Department of Orthodontics, University of Bonn, Welschnonnenstr. 17, 53111 Bonn, Germany; 2 Department of Oral Technology, University of Bonn, Welschnonnenstr. 17, 53111 Bonn, Germany; 3 Clinic of Orthodontics and Pediatric Dentistry, Center of Dental Medicine, University Zurich, Plattenstrasse 11, 8032 Zurich, Switzerland

**Keywords:** Prospective clinical study, Bite force, T‑Scan software, Retention protocols, Orthodontic retainers, Prospektive klinische Studie, Bisskraft, T‑Scan-Software, Retentionsprotokolle, Kieferorthopädische Retainer

## Abstract

**Purpose:**

Aim of the present study was to assess the relative distribution of occlusal forces after orthodontic treatment and during the first 3 months of the retention phase using a computerized occlusal analysis system (T-Scan, Tekscan Inc., Norwood, MA, USA).

**Materials and methods:**

A total of 52 patients were included in this prospective cohort study and underwent analysis of occlusal forces on the level of tooth, jaw-half, and -quadrant during a 3-month period. Furthermore, differences between three retention protocols (group I: removable appliances in both jaws; group II: fixed 3–3 lingual retainers in both jaws; group III: removable appliance in the maxilla and fixed 3–3 lingual retainer in mandible) were assessed with Wilcoxon signed-rank tests at 5%.

**Results:**

Directly after debonding, measured forces distribution were similar to published references for untreated samples. In the following, no significant difference was found between retention protocols II and III with regard to the asymmetry of the anterior occlusal forces. Both groups maintained an asymmetric force distribution in the anterior segment during the study period. There was also no difference between groups II and III in the distribution of occlusal forces for the posterior segments. Both retention concepts kept the symmetrical distribution of occlusal forces stable over the observation period. The retention concept of group I demonstrated a symmetrical distribution of occlusal forces in the anterior segment after debonding and this remained stable during the 3‑month period. In the posterior segment, no improvement of the initially asymmetric masticatory force distribution could be observed.

**Conclusions:**

All three studied retention protocols showed stability in retaining their original symmetrical or asymmetrical occlusal force distribution posteriorly/anteriorly during the 3‑month observation period. Therefore, an even distribution of occlusal forces should be the aim of the finishing phase, as no relative benefit of any single retention scheme in terms of post-debond improvement during the retention phase was seen.

## Abstract (de)

**Ziel der Studie:**

Ziel der vorliegenden Studie war es, die relative Verteilung der okklusalen Kräfte nach der kieferorthopädischen Behandlung und während der ersten 3 Monate der Retentionsphase mithilfe eines computergestützten okklusalen Analysesystems (T-Scan, Tekscan Inc., Norwood, MA, USA) zu untersuchen.

**Material und Methoden:**

52 Patienten wurden in die prospektive Kohortenstudie aufgenommen und es wurde über einen Zeitraum von 3 Monaten eine Analyse der Verteilung der okklusalen Kräfte bezogen auf die einzelnen Zähne, die Kieferhälften und die unterschiedlichen Quadranten vorgenommen. Darüber hinaus wurden die Unterschiede zwischen 3 Retentionsprotokollen (Gruppe I: herausnehmbare Apparaturen in beiden Kiefern; Gruppe II: festsitzender 3‑3-Lingualretainer in beiden Kiefern; Gruppe III: herausnehmbare Apparatur im Oberkiefer und festsitzender 3‑3-Lingualretainer im Unterkiefer) mithilfe des Wilcoxon-signed-rank-Tests bei einem Signifikanzniveau von 5 % bewertet.

**Ergebnisse:**

Unmittelbar nach dem Debonding waren die gemessenen Kräfteverteilungen ähnlich wie in bereits veröffentlichten Studien für unbehandelte Probanden. Im Folgenden wurde kein signifikanter Unterschied zwischen den Retentionsprotokollen II und III in Bezug auf eine Asymmetrie der anterioren okklusalen Kräfte festgestellt. Beide Gruppen behielten während des Studienzeitraums eine asymmetrische Kraftverteilung im Frontzahnbereich bei. Auch bei der Verteilung der Okklusionskräfte im Seitenzahnbereich gab es keinen Unterschied zwischen den Gruppen II und III. Beide Retentionskonzepte hielten eine symmetrische Verteilung der okklusalen Kräfte über den Beobachtungszeitraum stabil. Das Retentionskonzept der Gruppe I wies im Frontzahnbereich nach dem Debonding eine symmetrische Verteilung der Okklusionskräfte auf, die über den Dreimonatszeitraum stabil blieb. Im Seitenzahnbereich konnte keine Verbesserung der initialen asymmetrischen Kaukraftverteilung beobachtet werden.

**Schlussfolgerungen:**

Alle 3 untersuchten Retentionsprotokolle zeigten eine stabile Beibehaltung ihrer ursprünglichen symmetrischen oder asymmetrischen Verteilung der okklusalen Kräfte im Seitenzahnbereich bzw. im Frontzahnbereich während des 3‑monatigen Beobachtungszeitraums. Daher sollte eine gleichmäßige Verteilung der okklusalen Kräfte das Ziel bereits der Finishing-Phase der aktiven Therapie sein. Bezüglich der einzelnen Retentionsschemata zeigte keines der untersuchten Protokolle einen relativen Vorteil in Bezug auf mögliche Verbesserung der Kräfteverteilung während der Retentionsphase.

## Introduction

Occlusion is important in the planning of prosthetic, conservative and orthodontic treatment. As early as the 1970s, Andrews investigated in his work “Six keys to normal occlusion” the characteristics of a balanced occlusion and identified six essential characteristics that should represent the goal of every orthodontic or dental treatment [7].

An unbalanced occlusion with eccentric early contacts might be associated with damage to the tooth structure, periodontal adverse effects or, in the worst case, longitudinal root fractures [29]. Furthermore, incorrect loading might cause failure of prosthetic restorations, such as premature implant loss, and ceramic fractures or chipping [55]. There is also controversy in the literature about the role of occlusion in the development of craniomandibular dysfunction, even though evidence for a direct causal relationship between the two is missing [34, 38, 56].

A balanced occlusion is likewise among the goals of orthodontic treatment that, in cases of complex malocclusions, is inevitably associated with abundant changes of occlusal contacts. As Clark and Evans noted, orthodontic treatment fundamentally alters static and functional occlusal relationships [10] and can even inadvertently lead to occlusal discrepancies [44]. The goal of orthodontic therapy should be to establish a static occlusion without premature contacts, an even distribution of forces on all teeth, and a symmetrical distribution of forces on the right and left half of the jaw [12]. In addition, a balanced occlusion should be strived for, as is believed to be the key for a long-term stable treatment outcome [28].

However, it is a matter of discussion whether active orthodontic treatment should be prolonged to ensure that ‘ideal’ occlusal contacts are achieved [10], while the long-term relapse in cases of non-“ideal” post-orthodontic occlusions remains unclear [10]. On the other hand, it is mostly expected that during the initial period after removal of the fixed appliances the occlusion “settles” and occlusal contacts spontaneously improve during the retention phase [8, 14, 15, 47, 54]. Furthermore, some studies have already demonstrated that selection of an appropriate retention appliance might positively influence this settling process [23, 49, 50, 57]. The question therefore also arises whether the distribution of occlusal forces during the retention phase might be influenced by the selection of a specific retention concept.

Thus, the primary aim of the present study was to determine the distribution of occlusal forces after completed orthodontic treatment and to assess it during the first 3 months of the retention phase using a computer-controlled occlusal analysis system (T-Scan, version 9.1, Tekscan Inc., Norwood, MA, USA). The secondary aim was to assess any differences in terms of force distribution during a 3-month study period between the three different retention protocols.

The working hypotheses were (1) that removable appliances would hinder occlusal settling in the posterior region and, thus, prevent the settling of an equilibrated posterior force distribution; (2) that fixed retainers should favor vertical settling of the posterior teeth and make the distribution of occlusal forces more equilibrated in the posterior region, while preventing the settling of a more even distribution of occlusal forces anteriorly; and (3) that combinations of maxillary plates and mandibular retainers would allow vertical tooth movements both anteriorly and posteriorly and prove to be advantageous in terms of an overall balanced force distribution.

## Materials and methods

### Patient sample

An ethics committee vote for the present prospective cohort nonpharmacological clinical study was granted from the ethics committee of the Medical Faculty of the University of Bonn (No. 203/19). Written consent to participate in the study was obtained from all participants or their legal guardians. Included in this study were patients who successfully finished orthodontic treatment using fixed appliances at the Orthodontic Department of Bonn, who were male or female, over 12 years of age, who presented without medical contraindications, in the permanent dentition up to at least the second permanent molar, with an established Angle class I in the molar and canine region, having regular overjet/overbite with anterior tooth contact, and having no major transversal/sagittal/vertical discrepancies. Excluded were patients with general medical diseases such as metabolic diseases, diseases of calcium metabolism, endocrine dysfunctions, syndromes, and patients under immunosuppression. At the start of treatment, patients had presented with a spectrum of malocclusions typical for practice (Angle class I, II, or III). Patients with and without a performed extraction of premolars were included in the study. All patients received purely orthodontic treatment without any surgical procedures or adjuncts.

Sample size calculation took place with support of the Institute of Medical Biometry, Informatics, and Epidemiology (IMBIE) of the Medical Faculty of Bonn. For this pilot study, due to missing reference data and to the approach to collect as much data as possible, a margin of error of 10% and a confidence level of 85% were predefined. This resulted in a necessary sample size of *n* = 51 subjects, i.e., 17 subjects for the three subgroups.

Thus, in total, a sample of 52 patients was included and evaluated. All patients had completed active fixed appliance treatment by postgraduate students of the department under direct supervision of an instructor during the years 2017–2020. Only cases whose treatment outcome corresponded to categories A/A+ according to the criteria of the Quality Commission of the German Orthodontic Society [13] were included. The retention phase was planned individually depending on the indication and individual patient need. The decision criteria for the insertion of a fixed retainer included the extent of the anterior crowding or the baseline tooth malalignment. The following three retention concepts were followed and compared in this study:

I. Removable appliances (“Schwarz plates”) in the maxilla and mandible,
II. Fixed 3–3 lingual retainers in the maxilla and mandible, and
III. Combination of a removable Schwarz plate in the maxilla and a fixed 3–3 lingual retainer in the mandible.

For the final analysis, the sample was composed of 17 patients in group I, 18 patients in group II, and 17 patients in group III.

### Study protocol

**Fig. 1:**
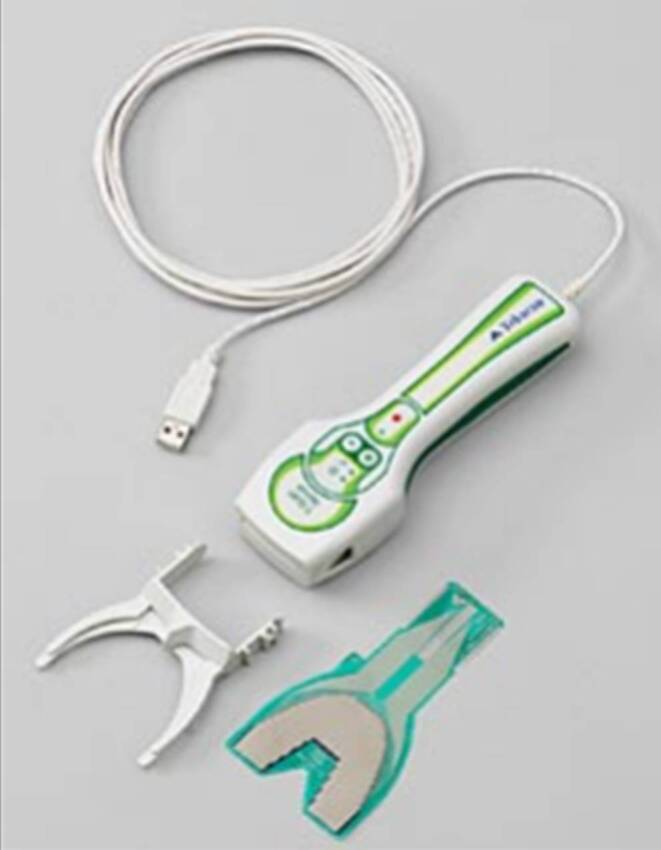
T‑Scan system (version 9.1), sensor handpiece (T-Scan Novus Handpiece DH‑1 System), sensor holder and sensor foil. (With kind permission © Cumdente GmbH, all rights reserved) T‑Scan-Software (Version 9.1), Sensorhandstück (T-Scan Novus Handpiece DH‑1 System), Sensorhalter und Sensorfolie. (Mit freundl. Genehmigung © Cumdente GmbH, alle Rechte vorbehalten)

**Fig. 2:**
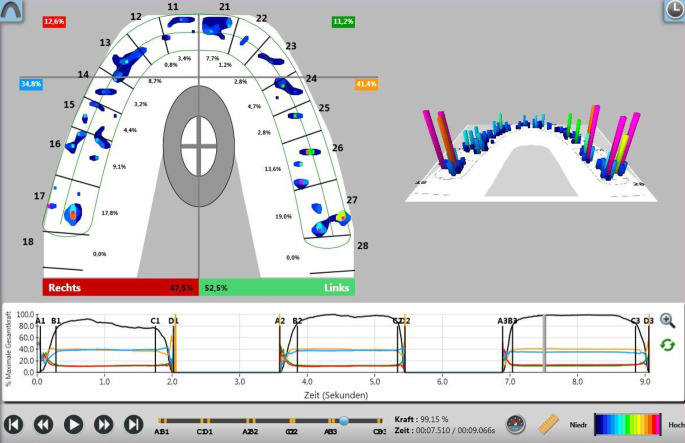
*Top left* In the two-dimensional diagram, the occlusal force distribution is visualized by contours and expressed in color or percentages. A coordinate system shows the distribution of the force levels in the four quadrants (left/right anterior, left/right posterior). *Top right* In the three-dimensional diagram, location and amount of force in the jaws are expressed as columns in different lengths and colors. *Below* Time/force diagram during three masticatory cycles (1–3). *A*: initial tooth contact; *B:* intercuspidation of all teeth during closure; *C:* beginning of disclusion; *D:* disclusion of all posterior teeth. (With kind permission © Cumdente GmbH, all rights reserved) *Oben links*: In der 2D-Grafik wird über ein Zahnbogenmodell die okklusale Kraftverteilung in Form von Konturen visualisiert und in Farbe bzw. Prozent ausgedrückt. Ein Koordinatensystem unterteilt die prozentuale Kraftverteilung auf die 4 Quadranten (links/rechts anterior, links/rechts posterior). *Oben rechts*: In der 3‑D Grafik werden über ein Zahnbogenmodell die Position und Kraftlevel der Okklusionskontakte als Säulen unterschiedlicher Höhe und Farbe visualisiert. *Unten*: Zeit/Kraft-Diagramm von 3 Kauzyklen (1–3). * A*: Initialer Zahnkontakt; *B:* Interkuspidation aller Zähne während des Mundschlusses; *C:* Beginn der Disklusion; *D:* Disklusion aller Seitenzähne. (Mit freundl. Genehmigung © Cumdente GmbH, alle Rechte vorbehalten)

Occlusal contacts and their distribution were measured using the T‑Scan System (Tekscan, Inc. Norwood, MA, USA) which consists of four components: the T‑Scan software (version 9.1), the sensor handpiece (T-Scan Novus Handpiece DH‑1 System, Cumdente, Tübingen, Germany), a sensor holder, and the sensor foil (Fig. 1). The T‑Scan software has different ways of displaying measured data. On the one hand, the occlusal contacts can be displayed two-dimensionally as a top view of the tooth row or three-dimensionally as a bar chart with columns of different heights (Fig. 2). This allows the relative force distribution of the occlusal contacts to be displayed. The relative force is indicated as a percentage of the maximum total occlusal force. Finally, the occlusal force that occurs during biting together can be displayed as a force–time diagram either for both jaw halves together or in a side-by-side comparison (right/left jaw half) separately from each other.

Measurements took place at three time points (T0, T1, T2) after completion of active orthodontic treatment. The first measurement took place on the day of debonding (T0). This measurement documented the baseline condition of the examination. During recording and calibration, the patient sat upright in the chair. The headrest was aligned so that the occlusal plane was parallel to the floor. Before starting the recording, the unit was calibrated to adjust the sensitivity of the system individually to the patient’s bite strength. The sensitivity level was set uniformly to an average. The calibration procedure is standardized by the software. The patient was first asked to bite the sensor foil once or twice to become acquainted with it and thus it was no longer consciously perceived by the patient. Then the patient bit down with maximum force and held the biting force for about 2–3 s until the automatic calibration was completed. The handpiece was then positioned again by means of a guide pin between the two central incisors, parallel to the occlusal plane on the maxillary arch. Pressing the record button started the T‑scan registration. The patient was asked to bite the foil three times with maximum force. Pressing the record button again ended the recording. Once the recording was complete, the occlusal contacts were displayed in two and three dimensions using the software. In addition, a force–time diagram provided a representation of the force development during clenching for the total force and also separately for the right and left half of the jaw. A total of three chewing cycles (multi-bite) were recorded per measurement. It was defined that at the time of maximum intercuspidation, the chewing force per tooth, per jaw half and per jaw quadrant was recorded and analyzed. The measured values were transferred to a spreadsheet.

In patients from group I, clear retainer splints (1 mm, Ekrolen transparent, Erkodent, Pfalzgrafenweiler, Germany) were inserted on this day, which were replaced by retention plates in the maxilla and mandible after about 10 days (T1). The retention plates included a labial bow, triangular clasps between the premolars, Adams claps on the first molar, and a resin base. In case of premolar extraction, the triangular clasps were omitted. Patients were instructed to wear their removable appliances 10–12 h per day.

In patients from group II, a 6-point fixed retainer made of triple-stranded super spring-hard wire (diameter 0.45 mm, Dentaurum, Ispringen, Germany) was bonded on the anterior teeth of the maxilla and mandible at T0 (Adhesive Primer/Bonding: Transbond XT Light Cure, 3M Unitek, St. Paul MN, USA; Composite: Kanisit Composite, Kaniedenta, Herford, Germany).

In patients from group III, a clear retainer was placed in the upper jaw at T0 which was replaced by a retention plate after about 10 days (T1) in the same way as in group I. In the mandible, in this group, a 6-point fixed retainer (see above) was inserted at T0, analogous to group II.

Measurement of occlusal contacts was repeated 10 days (T1) and 10 weeks (T2) after T0; this corresponds to about 3 months after debonding.

### Statistical analysis

Statistical analysis was carried out using the statistics program IBM SPSS Statistics 28.0 (IBM Corp., Armonk NY, USA). Testing for normal distribution of the data was performed using the Shapiro–Wilk test. Due to the lack of a normal distribution for most of the parameters, medians and interquartile ranges were calculated and all group comparisons were performed with the nonparametric Wilcoxon signed-rank test with a *p* ≤ 0.05 being considered as statistically significant. Due to the explorative character of the work, a correction of the *p*-values for multiple testing was waived.

## Results

### Patients

The mean age ± standard deviation (SD) of the entire patient sample (*n* = 52) at the time of debonding (T0) was 18.7 ± 4.0 years (group I: 18.4 ± 2.8 years, group II: 18.4 ± 4.7, group III: 19.3 ± 4.4 years). Of the total 52 patients, 29 (56%) were female (group I: 9 [53%]; group II: 9 [50%]; group III: 11 [67%]).

### Tooth-related data at the time of debonding

Table 1 shows the percentage distribution of occlusal forces for all upper teeth at the time of debonding (T0) for the entire patient sample (*n* = 52). The molars (teeth 17, 16, 26, 27) received the greatest occlusal forces with a median of 59.4%. In detail, tooth 17 received a median of 12.2%, tooth 16 of 14.0%, tooth 26 of 12.7%, and tooth 27 of 20.6% (Table 1). The anterior segment (teeth 12, 11, 21, 22) received significantly lower occlusal forces with a median of 8.6%. The lateral incisors (12, 22) received the smallest share of the total occlusal force (Table 1). The canines (13, 23) received a median of 7.8%, while the premolars (14, 15, 24, 25) received a median of 23.5% of the occlusal forces, (Table 1).

**Table 1:** Percentage distribution of occlusal forces for all upper teeth at debonding (T0) for all patients (*n* = 52) Prozentuale Verteilung der okklusalen Kräfte aller Zähne (17–27) zum Zeitpunkt der Entbänderung (T0) für das gesamte Patientenkollektiv (*n* = 52)

Tooth	Missing	Median (IQR)	Range
11	0/52	2.80 (1.43–6.60)	0–17.80
21	0/52	3.50 (1.25–7.73)	0–16.20
12	0/52	1.25 (0.25–2.55)	0–6.70
22	0/52	1.00 (0.05–2.48)	0–6.90
13	0/52	4.20 (2.35–7.85)	0–17.10
23	0/52	3.55 (2.13–5.40)	0.40–17.70
14	22/52	5.75 (2.38–9.13)	0.20–19.70
24	22/52	6.70 (3.45–9.10)	1.20–12.10
15	0/52	5.45 (2.85–8.20)	0–17.70
25	0/52	5.50 (2.63–8.28)	0–14.80
16	0/52	14.00 (10.05–16.05)	0–32.10
26	0/52	12.65 (6.75–16.65)	0–31.30
17	0/52	12.15 (6.70–17.63)	0–32.20
27	0/52	20.55 (14.13–25.48)	0.60–44.80

*IQR* interquartile range

Table 2 shows the relative force distribution between the right and left jaw halves at the time of debonding (T0) for the total sample (*n* = 52), with similar median values for the right (47.4%) and the left half of the jaw (52.7%). Thus, at the time of debonding (T0), i.e., after completion of active orthodontic therapy, the average exercised force was distributed almost equally between the two jaw halves.

**Table 2:** Percentage distribution of occlusal forces between jaw halves (right/left) and the quadrants (right/left and anterior/posterior) at debonding T0 (*n* = 52) Prozentuale Verteilung der okklusalen Kräfte zwischen den Kieferhälften rechts und links und den 4 Quadranten zum Zeitpunkt der Entbänderung T0 (*n* = 52)

Segment	Side	*n*	Median (IQR)	Range
Jaw half	Right	52	47.35 (42.35–52.30)	23.10–65.60
	Left	52	52.65 (47.70–57.43)	34.40–76.90
Quadrant	Right anterior	52	9.55 (6.15–13.45)	0.60–29.80
	Left anterior	52	8.25 (3.90–13.75)	0.40–18.20
	Right posterior	52	38.60 (31.43–43.40)	4.90–53.30
	Left posterior	52	42.55 (36.88–50.28)	25.40–73.90

*IQR* interquartile range

### Quadrant-related data at the time of debonding

The dentition was divided into anterior quadrants (including central/lateral incisors and canines) and posterior quadrants (including premolars and molars) (Fig. 2). Table 2 shows the relative distribution of occlusal forces for the two jaw halves and the four quadrants at the time of debonding (T0) for the total sample (*n* = 52). At debonding, the right anterior quadrant received a median of 9.6% of the occlusal forces and similar values (8.3%) were observed for the left anterior quadrant. The right posterior quadrant received a median of 38.6% of the occlusal forces, whereas the left posterior quadrant received a median of 42.6% (Table 2). Thus, at the time of debonding (T0), i.e., after completion of active orthodontic therapy, the total occlusal forces of the jaw were distributed approximately 20% versus 80% between the two anterior quadrants (sum of anterior quadrants right/left) and the two posterior quadrants (sum of posterior quadrants right/left), respectively. This data indicates that occlusal forces are not uniformly distributed (at around 25%) in the four mouth quadrants.

### Occlusal forces, related to the jaw-halves per time point and group

Table 3 shows the relative distribution of occlusal forces between the right/left half of the jaw at the three examination times T0, T1, and T2 and in the respective retention groups (I–III; Table 3). For group I, a statistically significant asymmetrical distribution of the occlusal forces was seen between the two halves of the jaw at time points T0 and T2 (Table 3). It can be concluded that in group I the distribution of occlusal forces between the right and left halves of the jaw did not change during the 3‑month study period (T2–T0).

**Table 3:** Percentage distribution of occlusal forces between jaw halves (left/right) at the three examination times T0, T1, and T2 and in the respective retention groups I–III (*n* = 52; no missing measurements) Prozentuale Verteilung der okklusalen Kräfte zwischen rechter und linker Kieferhälfte zu den Untersuchungszeitpunkten T0, T1 und T2 in den jeweiligen Retentionsgruppen I–III (*n* = 52; keine fehlenden Messungen)

Time	Group	Group	n	Median (IQR)	Range	*P*^a^
T0	I	Right half jaw	17	45.00 (42.30–48.50)	36.90–60.00	0.004
		Left half jaw	17	55.00 (51.50–57.70)	40.00–63.10	
	II	Right half jaw	18	50.30 (42.70–53.55)	33.00–61.10	0.60
		Left half jaw	18	49.70 (46.45–57.30)	38.90–67.00	
	III	Right half jaw	17	49.60 (41.50–56.80)	23.10–65.60	0.59
		Left half jaw	17	50.40 (43.20–58.50)	34.40–76.90	
T1	I	Right half jaw	17	45.90 (43.25–51.80)	37.30–64.20	0.16
		Left half jaw	17	54.10 (48.20–56.75)	35.60–62.70	
	II	Right half jaw	18	51.80 (47.55–55.18)	42.80–58.30	0.24
		Left half jaw	18	48.20 (44.83–52.45)	41.70–57.20	
	III	Right half jaw	17	50.40 (43.60–55.25)	34.50–63.20	0.85
		Left half jaw	17	49.60 (44.75–56.40)	36.80–62.50	
T2	I	Right half jaw	17	45.10 (40.00–50.20)	31.50–62.80	0.04
		Left half jaw	17	54.60 (49.80–60.00)	37.20–68.50	
	II	Right half jaw	18	50.40 (48.64–55.20)	38.80–62.80	0.33
		Left half jaw	18	49.60 (44.80–51.35)	37.20–61.20	
	III	Right half jaw	17	51.10 (41.45–53.85)	37.80–58.50	0.80
		Left half jaw	17	48.90 (46.15–58.55)	41.50–62.20	

*IQR* interquartile range

^a^2‑tailed asymptomatic value from Wilcoxon signed-rank test

For groups II and III, the statistical testing showed no statistically significant differences in the distribution of the occlusal forces between the right and left jaws at all three time points (Table 3). This means that at all time points (T0, T1, T2) there was a relatively symmetrical occlusal force distribution. It can be concluded from this that the retention concepts in groups II and III were associated with neither an improvement nor a deterioration of the result achieved through treatment over the 3‑month period. Table 4 shows the statistical comparison of occlusal force distribution of the right/left jaw half per group (I–III) and time intervals T1–T0, T2–T0, T2–T1. In all the time intervals, the distribution of the occlusal forces between the right and left remained stable in all groups (Table 4). Thus, overall the retention concept did not have a significant influence on the occlusal force distribution in any of the three groups and at any of the three examination times. With all three retention concepts, the distribution of the occlusal forces to the two jaw halves was stabilized.

**Table 4:** Statistical testing of occlusal force distribution changes over time by jaw half (right/left) and quadrant (right/left and anterior/posterior) in retention groups I–III and time intervals T1–T0, T2–T0, T2–T1 (*n* = 52) Statistische Untersuchung zum Vergleich der okklusalen Kräfte über die Zeit bezüglich der Kieferhälften rechts und links, der Quadranten anterior rechts, anterior links, posterior rechts und posterior links in den Retentionsgruppen I–III und in den Zeitintervallen T1–T0, T2–T0, T2–T1 (*n* = 52)

		*P*^a^		
Group	Segment	T1–T0	T2–T0	T2–T1
I	Right half jaw	0.06	0.91	0.32
	Left half jaw	0.06	0.91	0.32
	Right anterior quadrant	0.04	0.49	0.18
	Left anterior quadrant	0.04	0.52	0.49
	Right posterior quadrant	0.80	0.73	0.88
	Left posterior quadrant	0.01	0.83	0.23
II	Right half jaw	0.08	0.11	0.60
	Left half jaw	0.08	0.11	0.60
	Right anterior quadrant	0.21	0.27	0.03
	Left anterior quadrant	0.98	0.07	0.02
	Right posterior quadrant	0.36	0.06	0.04
	Left posterior quadrant	0.04	0.36	0.35
III	Right half jaw	0.28	0.76	0.65
	Left half jaw	0.28	0.76	0.65
	Right anterior quadrant	0.03	0.42	0.08
	Left anterior quadrant	0.96	0.16	0.18
	Right posterior quadrant	0.62	0.55	0.38
	Left posterior quadrant	0.22	0.96	0.22

^a^2‑tailed asymptomatic value from Wilcoxon signed-rank test

### Results related to the anterior jaw quadrants per time point and group

Table 5 shows the relative distribution of the occlusal forces between the two anterior quadrants (anterior right/left) at the three examination time points T0, T1, and T2 in the respective retention groups I–III. In group I, it was not possible to distinguish between the occlusal forces in the right and left anterior quadrants at any of the three time points. At the time of debonding (T0), there was an even distribution of the masticatory force between the two anterior quadrants, which remained constant over the entire study period (T2–T0) of approximately 3 months.

**Table 5:** Percentage distribution of the occlusal forces between the two anterior quadrants (right/left) at the three examination time points in the retention groups I–III (*n* = 52 patients; no missing measurements) Prozentuale Verteilung der okklusalen Kräfte zwischen rechten und linken anterioren Quadranten zu den Untersuchungszeitpunkten T0, T1 und T2 in den jeweiligen Retentionsgruppen I–III (*n* = 52; keine fehlenden Messungen)

Time	Group	Quadrant	*n*	Median (IQR)	Range	P^a^
T0	I	Right anterior	17	7.60 (3.80–10.95)	1.90–19.30	0.59
		Left anterior	17	8.20 (3.85–11.70)	0.40–16.00	
	II	Right anterior	18	11.00 (7.78–12.95)	0.60–18.80	0.04
		Left anterior	18	8.10 (6.55–14.10)	0.60–18.20	
	III	Right anterior	17	7.20 (6.05–21.55)	2.60–29.80	0.04
		Left anterior	17	10.00 (3.30–16.50)	2.90–17.60	
T1	I	Right anterior	17	9.00 (7.10–13.20)	0–24.50	0.93
		Left anterior	17	10.00 (6.05–11.85)	1.30–15.50	
	II	Right anterior	18	11.10 (6.35–17.70)	0–26.20	0.04
		Left anterior	18	8.20 (5.65–14.83)	0.30–17.90	
	III	Right anterior	17	10.90 (6.60–22.40)	1.30–34.10	0.03
		Left anterior	17	7.80 (2.80–17.25)	1.60–24.70	
T2	I	Right anterior	17	9.20 (3.85–12.90)	0–19.00	0.98
		Left anterior	17	8.00 (3.85–12.90)	2.30–17.30	
	II	Right anterior	18	8.60 (5.95–14.18)	0–20.00	0.04
		Left anterior	18	7.90 (3.03–11.78)	0–23.40	
	III	Right anterior	17	9.90 (5.05–20.65)	2.40–29.60	0.002
		Left anterior	17	9.70 (2.55–12.65)	0–24.60	

*IQR* interquartile range

^a^2‑tailed asymptomatic value from Wilcoxon signed-rank test

In group II, however, for the distribution between the right and left side, a significant difference was found for the anterior quadrants at all three timepoints (Table 4). During the observation period, no improvement or adjustment of the equilibrium between the right and left anterior quadrants took place. This means that with the help of the retention concept the treatment result remained stable during the entire observation period of about 3 months.

In group III, at all three timepoints a significant difference between the right and left anterior quadrants was observed (Table 4). At debonding (T0), a significant difference between the right and left anterior quadrants was present. As in group II, there was no improvement in the occlusal force distribution. This means that the retention concept of group III also stabilized the distribution of the occlusal forces between the right and left anterior quadrants.

### Results related to posterior jaw quadrants per timepoint and group

Table 6 shows the relative distribution of occlusal forces between the two posterior quadrants (posterior right/left) at the three study timepoints T0, T1, and T2 in the respective retention groups.

**Table 6:** Percentage distribution of the occlusal forces between the two posterior quadrants (right/left) at the three examination timepoints in retention groups I–III (*n* = 52 patients; no missing measurements) Prozentuale Verteilung der okklusalen Kräfte auf die posterioren Quadranten zu den Zeitpunkten T0, T1 und T2 in den Retentionsgruppen I–III (*n* = 52; keine fehlenden Messungen)

Time	Group	Quadrant	*n*	Median (IQR)	Range	*P*^a^
T0	I	Right posterior	17	35.50 (31.35–42.70)	28.60–53.30	0.005
		Left posterior	17	44.70 (40.00–50.85)	35.70–58.80	
	II	Right posterior	18	40.90 (31.20–44.55)	21.20–50.30	0.29
		Left posterior	18	40.10 (36.00–49.85)	29.00–58.70	
	III	Right posterior	17	39.50 (29.80–43.85)	4.90–49.80	0.23
		Left posterior	17	46.50 (28.75–50.70)	25.40–73.90	
T1	I	Right posterior	17	35.10 (31.75–42.35)	24.00–52.10	0.04
		Left posterior	17	42.60 (40.40–47.30)	29.80–57.10	
	II	Right posterior	18	39.60 (33.65–45.33)	24.20–51.10	0.85
		Left posterior	18	37.50 (34.23–48.10)	25.80–51.60	
	III	Right posterior	17	36.00 (26.85–42.25)	13.80–50.70	0.19
		Left posterior	17	39.80 (26.45–52.65)	20.30–59.00	
T2	I	Right posterior	17	37.10 (31.05–42.15)	20.50–50.00	0.01
		Left posterior	17	48.90 (37.70–52.40)	34.40–57.70	
	II	Right posterior	18	41.45 (37.23–47.90)	24.40–52.10	0.71
		Left posterior	18	39.85 (34.58–47.73)	27.00–51.50	
	III	Right posterior	17	37.30 (34.10–42.20)	18.80–46.20	0.16
		Left posterior	17	41.10 (32.60–53.45)	16.90–59.80	

*IQR* interquartile range

^a^2‑tailed asymptomatic value from Wilcoxon signed-rank

In group I, regarding the distribution of occlusal forces between the right and left posterior quadrants, a significant difference was found at all three timepoints (Table 4). This situation remained constant over the observation period (T0–T2). Thus, the treatment outcome achieved by active orthodontic treatment did not change significantly during the initial retention period of 3 months if this retention protocol was followed.

In group II, there were no differences in the distribution of the occlusal force between the right and left posterior quadrants at the three timepoints (Table 4). The uniform distribution between the two posterior quadrants remained constant throughout the study period.

In group III, there was no difference in the distribution of the occlusal forces between the right and left posterior quadrants at the three timepoints (Table 6). In the side-by-side comparison, the symmetrical distribution could be stabilized during the study period.

In Table 4, time-dependent changes of the occlusal force distribution of the different regions of the jaws (T1–T0, T2–T0, T2–T1) are presented. Comparing the anterior right/left jaw quadrants over the time interval T2–T0 found no significant change in the occlusal force distribution for all three groups. In the overwhelming majority of cases, no changes could be detected. Thus, in general, in all three retention groups, the distribution of the occlusal forces in the two anterior quadrants remained stable during the 3‑month study period. In detail, removable appliances maintained a symmetrical force distribution in the anterior quadrant (group I). After insertion of a fixed retainer, either in both jaws (group II) or in only one jaw, (group III) any asymmetric force distribution at the end of the active treatment phase did not seem to improve in the course of the examination period. With regard to the distribution of the occlusal forces on the posterior right and left jaw quadrants over the time interval T2–T0, very few statistically significant changes were found in the three groups. Thus, in all three retention groups, the distribution of the occlusal force between the two posterior quadrants remained relatively stable. Removable appliances maintained an uneven distribution in the posterior region and prevented a symmetrical adjustment of occlusal forces (group I). The retention concepts of group II and III maintained a symmetrical occlusal force distribution during the 3‑month study period.

## Discussion

The aim of the present study was to assess the distribution of occlusal forces after completion of orthodontic treatment and to document any changes during the first 3 months of the retention phase using a computerized occlusal analysis system (T-Scan, version 9.1).

### Discussion of the methodology and limitations of the study

It can be critically noted that a short observation period of only 3 months was covered in the present study, which was however, based on data from the literature. Bauer et al. found that the greatest increase in occlusal contacts during settling can be expected in the first 2 months after debonding [8], which is also reflected in other studies utilizing an observation period of 3 months [15, 57]. The present study did not include a control group without retention devices for ethical reasons, as post-orthodontic relapse in the absence of any stabilizing appliance can be expected [25, 32]. Furthermore, no objective assessment of patient compliance for wear of the removable appliances using wear time sensors took place, which introduces uncertainty as to the actual wear time of the prescribed appliances.

With respect to the analysis of the occlusal contacts and forces, regardless of which variant is used to represent the occlusal contacts, it must be taken into account that individual factors can influence these measurements. Among other things, tooth mobility and its change during a recurring load within the alveolus can distort the measured result. The load angle/force vector depending on mandibular movement or any twisting of the mandible, even with jaw closure, can also distort the measured result. Carey et al. were able to show in their study that each of these individual factors can introduce noise in the measurement process and affect the significance of a statistical analysis [9].

Regarding the reproducibility of the T‑Scan system, the main possible source of error in the determination of occlusal contacts could be the foil thickness which is specified at 65 μm [52]. Halperin et al. recommended a layer thickness between 13–21 μm for occlusal strips, so that they are below the physiological occlusal perception level of most patients [19]. There is a definite risk that the patient will perceive the strip as an interfering contact and will adopt a different mandibular position for proprioceptive reasons [19]. Another weakness of the T‑Scan system is the standardized dental arch model, which does not always correspond to the individual patient-specific dental arch shape. Although the dental arch model can be adjusted to some extent by entering the mesiodistal dental arch width of the upper incisor, small deviations can lead to difficulties in accurate contact point localization [21, 36, 42]. Another possible source of error is the identical positioning of the sensor foil during repeated measurements. Although the incisive mandrel serves as a rough positioning aid, it is still possible to twist the sensor holder with the foil clamped in place. On the other hand, in recent years, T‑Scan technology has been continuously developed in the area of sensor foil technology and software, so that the described sources of error could be minimized [46, 52]. In their study, Koos et al., analyzing the measurement accuracy and reliability of the T‑Scan III system, came to the conclusion that acceptable measurement accuracy exists. Interfering influences due to changing foils or repeated measurements could not be observed [27]. Recent studies also demonstrated that the precision and reproducibility in repeated measurements were also excellent [30, 46].

### Occlusal force distribution across teeth, jaw-halves, and quadrants at time T0

Regarding the individual occlusal forces per tooth, the present study found that at the time of debonding, the molars received the greatest part of occlusal forces (median = 59.4%) and the incisors received the smallest (median = 8.6%). At the same time, immediately after active orthodontic therapy, the occlusal forces were distributed almost equally between the two jaw halves (right 47.4% and left 52.7%). Furthermore, occlusal forces were distributed in a 20% vs 80% relation to the two anterior and the two posterior quadrants, respectively.

Comparing the results of this study with others, some similarities can be found in terms of the individual force distribution per tooth, jaw-half, and quadrant.

Alkan et al. [4] used the T‑Scan III system to investigate the individual distribution of occlusal contacts per tooth after completion of orthodontic treatment and found similar results to the present study, as the molars received the greatest part of occlusal forces, and the incisors received the smallest part. In addition, Alkan et al. [4] reported similar results with the present study regarding the force distributions according to individual teeth, jaw-halves, or quadrants.

Different studies analyzed the occlusal force distribution of either untreated populations or orthodontically treated patients using the T‑Scan system. In general, orthodontically treated populations showed similar force distributions to untreated populations [16, 24, 37, 39, 41]. In the right/left side comparison, an equal distribution was observed, and the two anterior quadrants typically received lower occlusal forces than the two posterior quadrants [1, 3, 4, 35, 44, 45]. Ma et al. investigated the occlusal force distribution in 53 subjects with neutral occlusion with the T‑Scan III system and found at maximum intercuspidation similar absolute values for the individual teeth and a similar distribution pattern to the present [35]. In contrast, An et al. found that patients after orthodontic treatment exerted stronger occlusal forces on the anterior teeth compared to an untreated control group [6].

### Settling processes during the retention phase

It is well established that some tooth movement occurs during the retention phase after the removal of the fixed appliances. A number of authors have already dealt with these occlusal changes during the orthodontic retention phase, which can be attributed to the settling process [8, 14, 15, 47, 54]. Due to the physiological intrinsic mobility of teeth in their socket and the ability to extrude, a spontaneous increase in the number of occlusal contacts after removal of the fixed appliance has been reported [53]. However, although studies report increased contacts during settling, the magnitude and extent of this increase was unpredictable [18]. This increase in occlusal contacts appears to mostly take place during the first 2 months but seems to continue up to 21 months after debond [8, 17, 47], and the influence of the retention scheme is unclear.

He et al. using the T‑Scan II system found an improvement in dynamic occlusion during the first year after debond and concluded that an assessment of occlusal forces and their distribution should be mandatory prior to debonding [20]. Cohen-Levy and Cohen [11] using the T‑Scan III system likely came to similar conclusions, as any uneven force distributions seen at debond remained during the retention phase. Similar findings were found by Morton and Pancherz [40] who found small changes in functional occlusion on average 2 years after occlusion, with 44.3% of the patients finishing orthodontic treatment with nonsatisfactory occlusion, and occlusal parameters remaining stable in the vast majority (72.3%) of these patients Therefore, no spontaneous correction of occlusal contacts was demonstrated after debond and the desired occlusal contacts/forces should be checked before removing the fixed appliances [11, 20, 40, 44, 45].

### Influence of the retention appliance on occlusal force distribution

As far as differences in the different retention schemes are concerned, the present study found that retainer choice had no effect on the post-orthodontic force distribution between the right and left jaw halves or between the two anterior quadrants, which remained stable. Therefore, working hypothesis 2 could be confirmed, since the insertion of a fixed retainer led to a rigid stabilization of the anterior segment. Fixed retainers in both jaws (group II) did not allow any changes in the anterior region and prevented the setting of a balanced occlusal force distribution in this region. Thus, working hypothesis 3 was refuted regarding the anterior region. The assumption that vertical tooth movements are favored anteriorly if a combination of a removable appliance and a fixed retainer is applied (group III) could not be confirmed.

The retention concepts of groups II and III maintained the (unchanged) symmetrical occlusal force distribution during the 3‑month study period. Removable appliances maintained an uneven distribution in the posterior region and prevented a symmetrical adjustment of the occlusal forces (group I). Working hypothesis 1 could therefore be confirmed, too. A possible cause for this could be the interdental clasp portions of the retention plates, which held the posterior teeth in their vertical position and prevented the adjustment of a symmetrical distribution of forces in the posterior segments. Since group III already had a symmetrical posterior distribution at T0, no statement can be made about a possible improvement of the posterior force distribution with the combination of fixed lingual retainers and removable plate appliances. Thus, with regard to occlusal force distribution in the posterior region, working hypothesis 3 can neither be confirmed nor refuted.

In general, retention concepts I–III kept the treatment result stable, regardless of whether symmetrical or asymmetrical conditions were present at the end of the active treatment phase. In practice, it can be concluded that the choice of retention appliance has little influence on the distribution of the occlusal forces and a spontaneous improvement of an asymmetrical force distribution during the first 3 retention months cannot be expected.

Existing data in the literature do not support a uniform retention protocol after treatment [2, 5, 22, 32, 48] and data about dental development and occlusal contact change remain controversial [3, 4, 23, 33, 43, 49, 50, 57]. Often, direct comparison between various studies is difficult, due to different methodologies used to determine occlusal contacts, different devices, and observation periods. A common feature of all existing studies is their rather small sample size (usually 15–20 patients per study) and therefore, further studies with consistent methodology, longer follow-up, and larger sample sizes might be useful [2, 22, 31, 32, 48].

On the one hand, an increase of occlusal contacts, depending on the vertical tooth movement that the respective retention appliances allow, has been demonstrated by some studies [23, 49, 50, 57]. These reported that the selection of a particular retention appliance influenced the processes of settling during the retention phase, depending on the design of the appliance. An occlusal hold of the teeth, as in the case of retention plates or vacuum-formed splints over the entire dental arch, tended to inhibit the extent of vertical movements of the teeth (50, 57), while appliances without occlusal overlay tended to enable this vertical movement (23 and 50). Contrary to that, no difference in occlusal contacts’ change during the first 2 months of retention was found between wrap-around retainers and vacuum formed-splints and occlusal forces remained stable [33, 46]. Finally, Alkan and Kaya [3] assessing occlusal force changes 6 months after debond found that while vacuum-formed splints and the Hawley retainers retained occlusal force distributions, significant changes in the force distribution of patients with fixed retainers were seen.

Thus, so far, there is no clear evidence for a spontaneous improvement of occlusal force distribution during the retention phase. The same holds true for the hypothesis that it can be positively influenced by the selection of a specific retention device. Therefore, an even distribution of occlusal forces should be checked or corrected already in the finishing phase of active orthodontic treatment [20, 44, 51]. Thorough analysis of the occlusal contacts and of the distribution of the occlusal forces using a device like the T‑Scan during the finishing phase may facilitate the establishment of a balanced and harmonious occlusion with an even occlusal force distribution [12, 26].

### Conclusions

The results of the present study indicated that all three investigated retention protocols retained the asymmetrical or symmetrical occlusal force distribution that was observed at the time of debonding. Insertion of a lingual fixed retainer in both jaws (group II) prevented the development of a symmetrical distribution of the occlusal forces in the anterior region and removable plates in both jaws prevented this in the posterior region (group I). In the combined fixed–removable retention group (group III), no symmetrical adjustment of the force distribution in the anterior segment could be observed and, thus, the initial hypothesis of a relative benefit over the other retention schemes could not be supported. Further studies with a larger number of subjects, consistent methodology, and a longer observation period might be helpful in further elucidating possible benefits of a specific retention protocol in terms of post-orthodontic settling of the occlusion.
